# Simulation-based camera navigation training in laparoscopy—a randomized trial

**DOI:** 10.1007/s00464-016-5210-5

**Published:** 2016-10-21

**Authors:** Cecilia Nilsson, Jette Led Sorensen, Lars Konge, Mikkel Westen, Morten Stadeager, Bent Ottesen, Flemming Bjerrum

**Affiliations:** 1 Department of Obstetrics and Gynecology, The Juliane Marie Centre for Children, Women and Reproduction, Rigshospitalet, University of Copenhagen, Blegdamsvej 9, 2100 Copenhagen, Denmark; 2 Copenhagen Academy for Medical Education and Simulation, Copenhagen, Denmark; 3 Department of Surgery, Hvidovre Hospital, Copenhagen, Denmark

**Keywords:** Camera navigation, Virtual reality, Laparoscopic surgery, Surgical education, Simulator, Motivation

## Abstract

**Background:**

Inexperienced operating assistants are often tasked with the important role of handling camera navigation during laparoscopic surgery. Incorrect handling can lead to poor visualization, increased operating time, and frustration for the operating surgeon—all of which can compromise patient safety. The objectives of this trial were to examine how to train laparoscopic camera navigation and to explore the transfer of skills to the operating room.

**Materials and methods:**

A randomized, single-center superiority trial with three groups: The first group practiced simulation-based camera navigation tasks (camera group), the second group practiced performing a simulation-based cholecystectomy (procedure group), and the third group received no training (control group). Participants were surgical novices without prior laparoscopic experience. The primary outcome was assessment of camera navigation skills during a laparoscopic cholecystectomy. The secondary outcome was technical skills after training, using a previously developed model for testing camera navigational skills. The exploratory outcome measured participants’ motivation toward the task as an operating assistant.

**Results:**

Thirty-six participants were randomized. No significant difference was found in the primary outcome between the three groups (*p* = 0.279). The secondary outcome showed no significant difference between the interventions groups, total time 167 s (95% CI, 118–217) and 194 s (95% CI, 152–236) for the camera group and the procedure group, respectively (*p* = 0.369). Both interventions groups were significantly faster than the control group, 307 s (95% CI, 202–412), *p* = 0.018 and *p* = 0.045, respectively. On the exploratory outcome, the control group for two dimensions, interest/enjoyment (*p* = 0.030) and perceived choice (*p* = 0.033), had a higher score.

**Conclusions:**

Simulation-based training improves the technical skills required for camera navigation, regardless of practicing camera navigation or the procedure itself. Transfer to the clinical setting could, however, not be demonstrated. The control group demonstrated higher interest/enjoyment and perceived choice than the camera group.

Camera navigation in laparoscopy is often considered a simple task and is handled by the less experienced, such as medical students or junior residents. It is, however, a complicated task, requiring specific psychomotor and visuospatial skills. Inappropriate handling of the camera results in poor visualization, which can lead to longer operating time [1–3]; surgeon frustration; and can, most importantly, compromise patient safety [4].

It is widely accepted that simulation-based basic laparoscopic training is beneficial and can be used for both training and assessment to prepare future surgeons prior to operating on patients [5, 6]. Existing studies have shown that simulation-based camera navigation training is beneficial compared with no training when tested on simulators [7, 8] or in a porcine model [9, 10]. One randomized trial demonstrated that simulation-based training of camera navigation skills transferred to the operating room (OR) and was as effective as traditional hands-on training but more time-efficient [11]. It is unknown how to optimally structure a training program for operating assistants and whether training of the technical skills for camera navigation or training on the procedure itself is most beneficial. Potentially, knowledge of and hands-on practice with the procedure itself could result in greater understanding of the surgeon’s needs and increased intrinsic motivation toward performing well as an operating assistant. Research on motivation in medical education is scarce [12], but it is an important part of understanding the components of learning [13].

The objective of the trial was to examine whether skills as an operating assistant were transferable to the OR after training on one of two fundamentally different laparoscopic tasks and whether different types of training influence motivational factors. The hypothesis was that simulation-based training improves laparoscopic camera navigation skills in the OR and increases motivation toward the task as an operation assistant.

## Materials and methods

### Design

**Fig. 1:**
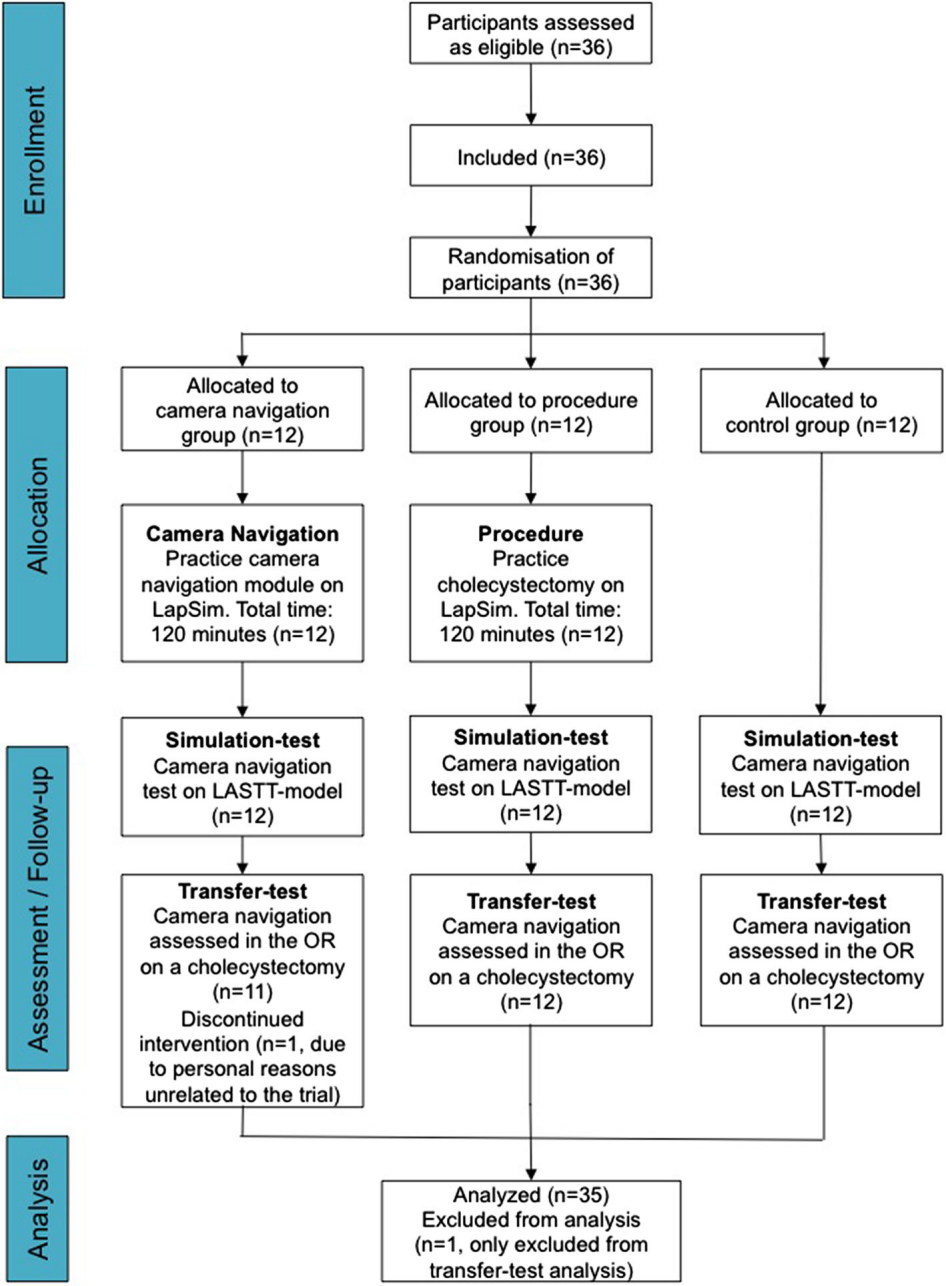
CONSORT-flowchart

A single-center randomized superiority trial was planned according to the CONSORT Statement (Fig. 1). The trial was exempt for ethical approval by the Regional Committee on the Biomedical Research Ethics (Ref. 15008637) and registered at ClinicalTrials.gov (NCT02530099) before inclusion of the first participant.

### Participants

Surgical novices were recruited through the student newspaper and student associations for general surgery and gynecology. The inclusion criteria were: (1) medical student in the fourth, fifth, or sixth semester, enrolled at the Faculty of Health Sciences at the University of Copenhagen, (2) informed consent for the trial. The exclusion criteria were: (1) previous participation in projects involving laparoscopic training, (2) experience with laparoscopic surgery (>0 procedures), and (3) not speaking Danish at a conversational level.

### Sample size calculation

The sample size was calculated based on the total score on the assessment tool (the primary outcome) used in the trial, with a difference of six points considered the minimally relevant difference. A standard deviation of four points was expected. With alpha set at 0.05 and a power of 0.90, and after adjusting for multiple comparisons, the total sample size required was 36 participants, 12 in each group.

### Randomization

A 1:1:1 randomization was performed centrally using a customized online Web-based system, Sealed Envelope (London, UK). The allocation sequence was random permuted blocks of 4, 6, or 8. The allocation sequence was kept concealed from the investigator during the trial. The randomization was stratified by sex (man/woman).

### The intervention

The trial was conducted at the Simulation Center at Rigshospitalet, University of Copenhagen [14]. The trial included two intervention groups and one control group, as shown in Fig. 1. The two interventions consisted of structured virtual reality simulation training for 120 min, with the task dependent on the assigned group. The first intervention group (camera group) practiced three different camera navigation tasks at three levels of difficulty, using a 30° angled laparoscope. The modules consisted of (1) finding stones in a virtual reality environment, and then focusing and aligning the camera on the stone, (2) localizing a specific gastrointestinal anatomical structure, and then focusing and aligning the camera, and (3) localizing a specific gynecological anatomical structure, and then focusing and aligning the camera. The second intervention group (procedure group) practiced a simulated laparoscopic cholecystectomy. The module consisted of dissecting and dividing the cystic duct and artery, followed by separation of the gallbladder from the liver. The principal investigator (CN) was present at all sessions, supervised training, and provided all feedback, which was given on request. Written instructions and instructional videos were available to the participants. The control group did not receive any training. Immediately after completion of training, the technical aspects of the camera navigation skills were tested using the Laparoscopic Skills Testing and Training (LASTT) model, after receiving standardized instructions. If assigned to the control group, the simulation test was performed immediately after randomization. After completion of the simulation test, participants were scheduled for the transfer test, which included assessment of camera navigation skills while assisting an outpatient cholecystectomy. One of two surgeons (MS, MW) performed or supervised all surgical procedures and completed all ratings. The same day as the transfer test, all participants received a questionnaire via email, exploring the motivation and perception of their role as an operating assistant and camera navigator. A 22-item version of the Intrinsic Motivation Inventory (IMI) was used, which is divided into four dimensions: interest/enjoyment, perceived competence, perceived choice, and pressure/tension [15].

### The transfer test and the assessment tool

The transfer test took place in the outpatient clinic at Hvidovre Hospital, Capital Region, Denmark. An assessment tool was created, inspired by the Objective Structured Assessment of Surgical Skills (OSATS) assessment tool, with five items scored on 5-point scales with anchors in the middle and at the ends [16]. The items were designed by a group consisting of the primary author (CN), two experienced laparoscopists (MW, MS), two senior researchers in assessment and medical education (JLS, LK), and a junior surgeon with experience in both laparoscopy and assessment research (FB). The final tool is given in Table 1.

**Table 1:**
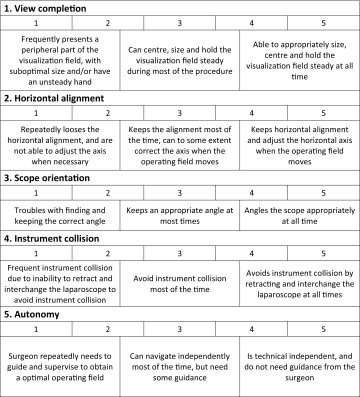
Objective structured assessment of camera navigation skills—OSA-CNS

### Simulator equipment

**Fig. 2:**
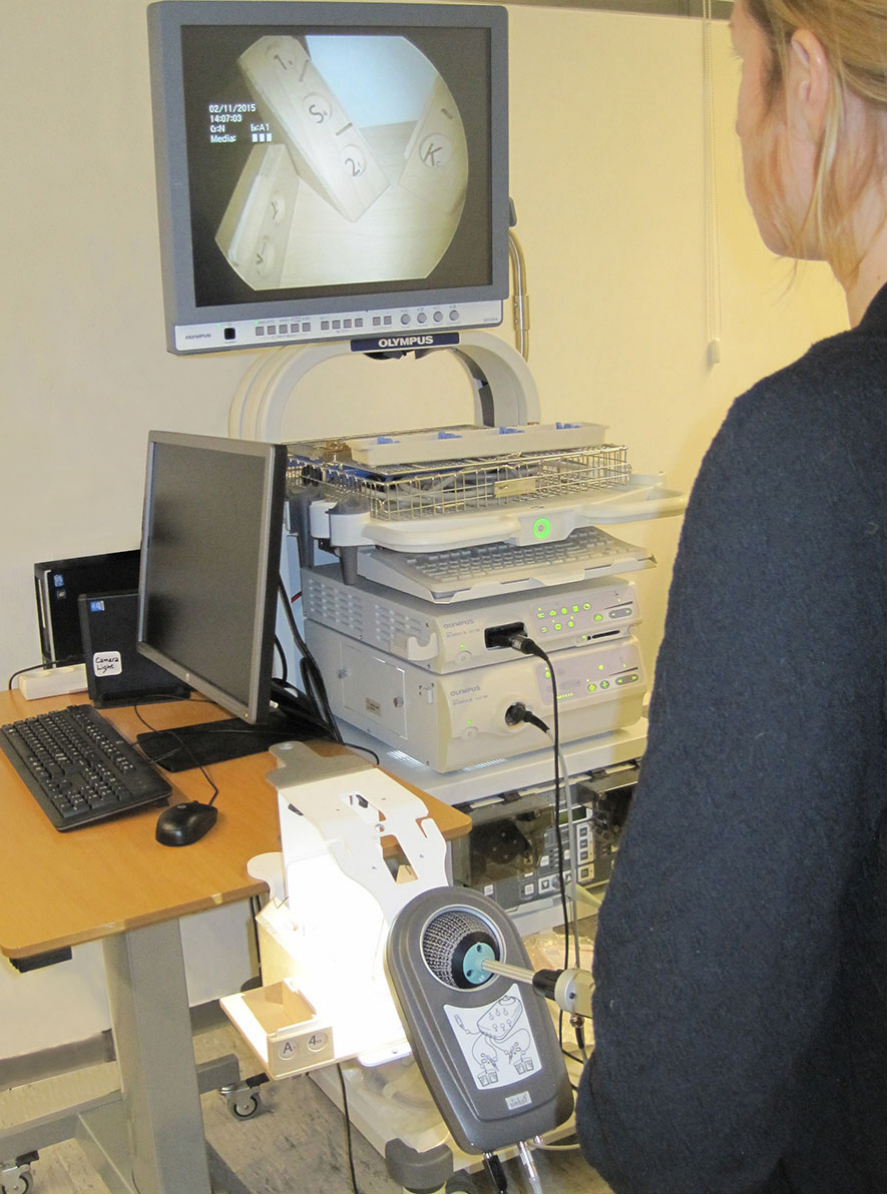
Simulation test setup

**Fig. 3:**
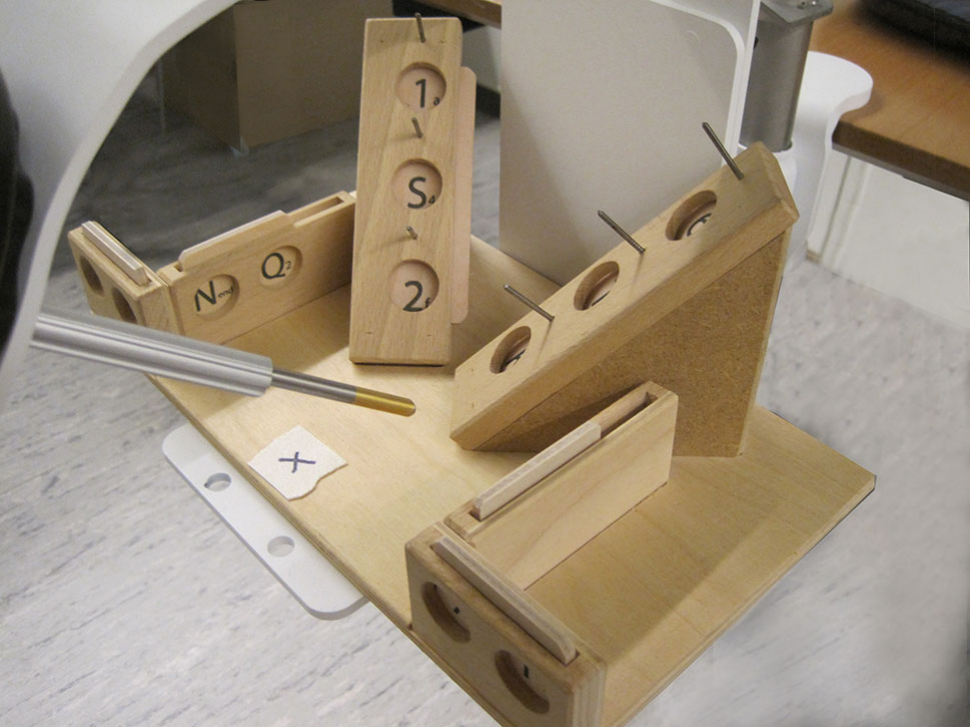
LASTT model

The LapSim^®^ virtual reality simulator (software version 2015) from Surgical Science (Göteborg, Sweden) generates a virtual operating field that can be viewed on a computer screen. Through the user interface, the participants interacted with the virtual operating field and performed the above-mentioned tasks. For the simulation test, the LASTT model [17, 18] was used with a 5-mm 30° angled laparoscope from Olympus (Tokyo, Japan) connected to a Simball 4D Joystick from G-coder Systems (Göteborg, Sweden), which records instrument movements and stores them on a computer (Fig. 2). The exercise tested the participant’s ability to navigate the laparoscope, while identifying 14 different targets placed at different sites in the LASTT model. Each target included a large symbol identifiable from a panoramic viewpoint and a small symbol only identifiable from a close-up viewpoint (Fig. 3). The targets were mounted such that they could only be identified by moving the laparoscope in different directions (rotation, lateral, and zoom-in/out). Three parameters were recorded: total time, total path length, and total angular path length.

### Outcomes

The primary outcome was the total score on the assessment tool, rated by the surgeon during a cholecystectomy. The secondary outcomes were motor skills parameters during the simulation-based test on the LASTT model (total time [seconds], total path length [centimeters], and total angular path length [degrees]). The exploratory outcome was the four dimensions (interest/enjoyment, perceived competence, perceived choice, and pressure/tension [reversed]) on the 22-item version of the IMI [15].

### Statistical analyses

Data were analyzed using SPSS version 22.0 (IBM, Armonk, New York, USA). All parameters were analyzed using one-way ANOVA with a two-sided significance level of *p* < 0.05. If a significant difference was observed, a group-wise comparison was performed.

Equality of variances was tested using Levene’s test, and depending on this, either Student’s *t* test or Welch’s *t* test was used. *p*-values of less than 0.05 were considered statically significant.

## Results

**Fig. 4:**
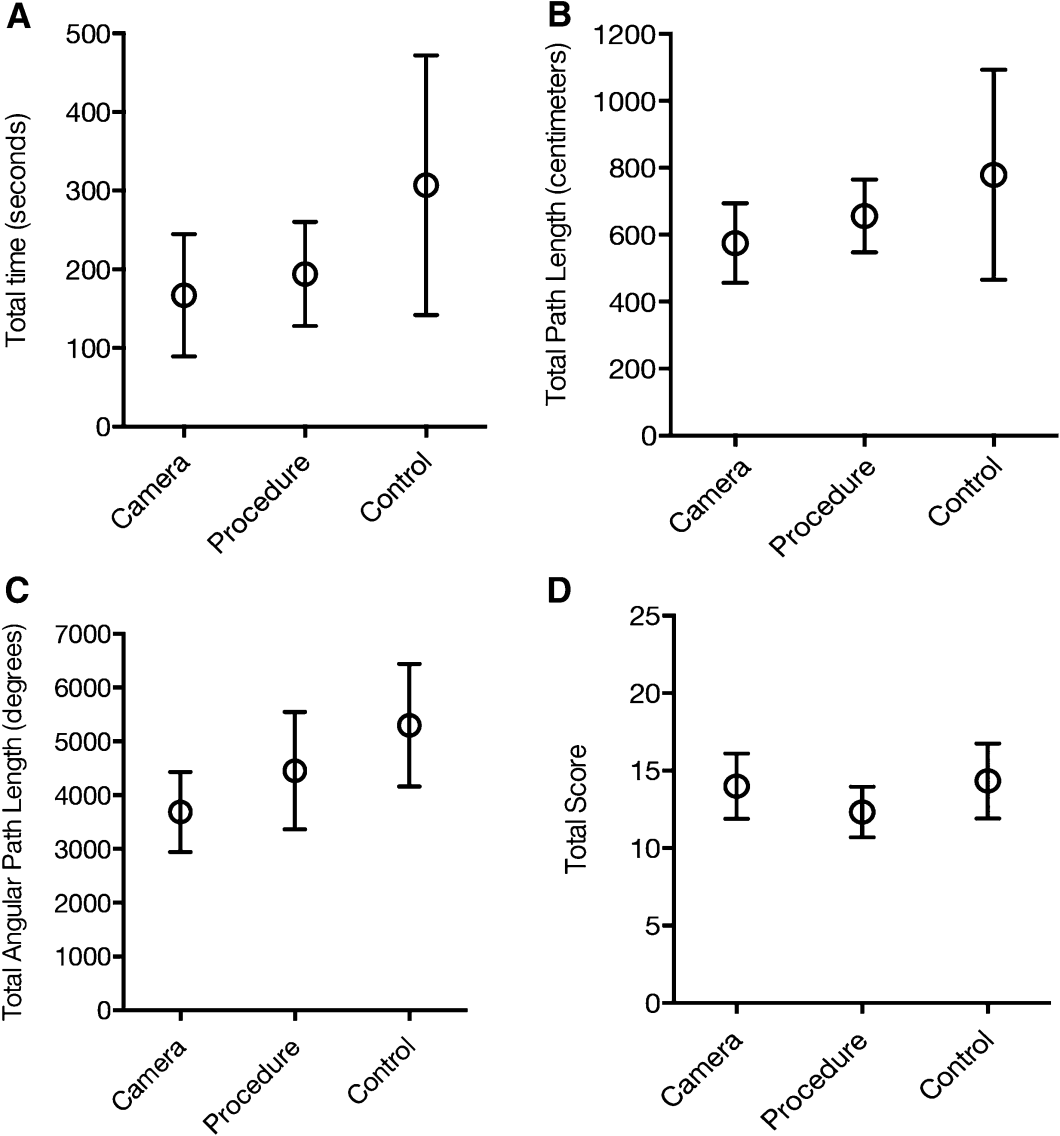
Plot A, B, and C demonstrating the motor skills measured during the simulation test. Plot D showing total score on the transfer test

A total of 36 participants were included and randomized. One participant dropped out due to personal reasons and could therefore not complete the transfer test and the IMI. The three groups’ baseline characteristics are given in Table 2. Significant differences between the groups were found on total angular path length (*p* = 0.027), total time (*p* = 0.010), and interest/enjoyment on the IMI (*p* = 0.039). No significant difference in total score on the transfer test was found between the groups (*p* = 0.279). The mean total score was 14.0 (95% CI, 11.9–16.1) for the camera group, 12.3 (95% CI, 10.7–14.0) for the procedure group, and 14.3 (95% CI, 11.9–16.6) for the control group. Pairwise comparison for the secondary outcome showed that total time on the LASTT model, the camera group (167 s; 95% CI, 117–216) was significantly faster (*p* = 0.018) than the control group (307 s; 95% CI, 202–412). The total angular path length was 3686 degrees (95% CI, 2943–4429) for the camera group versus 5300 degrees (95% CI, 4161-6441) for the control group (*p* = 0.016). Additionally, the total time for the procedure group was 194 s (95% CI, 152–236) versus 307 s (95% CI, 202–412) for the control group (*p* = 0.045). No significant differences between the camera group and the procedure group were observed (Fig. 4). For the exploratory outcome, a significant difference was found between the camera group and the control group on two dimensions, interest/enjoyment (*p* = 0.030) and perceived choice (*p* = 0.033). On interest/enjoyment, the camera group scored 5.5 (95% CI, 5.2–5.9) versus the control group 6.4 (95% CI, 5.9–6.9), indicating higher interest and enjoyment in the control group. On perceived choice, the camera group scored 6.3 (95% CI, 5.9–6.6) and the control group 6.7 (95% CI, 6.5–7.0), indicating a higher level of perceived choice. No significant difference was found on the dimensions of pressure/tension and perceived competence (Table 3).

**Table 2:** Participant baseline characteristics

	Camera group (*n* = 12)	Procedure group (*n* = 12)	Control group (*n* = 12)
Gender, (male: female)	8:4	4:8	3:9
Age, median (years), (interquartile range)	22 (21–23)	22 (22–23)	23 (21–24)
Time from intervention to transfer test (days), median (interquartile range)	13 (6–19)	11(5–21)	–

**Table 3:** Intrinsic Motivation Inventory (range 1–7). Mean (95% confidence interval) for motivation and perception toward the task as a camera navigator during a cholecystectomy

		*p*-value		
	IMI mean (95% CI)	Control versus camera	Camera versus procedure	Procedure versus control
Interest/enjoyment		0.03	0.07	0.44
Camera	5.5 (5.2–5.9)			
Procedure	6.2 (5.8–6.6)			
Control	6.4 (5.9–6.9)			
Perceived competence		0.50	0.44	0.84
Camera	5.0 (4.5–5.5)			
Procedure	5.3 (4.7–5.8)			
Control	5.2 (4.8–5.6)			
Perceived choice		0.03	0.33	0.25
Camera	6.3 (5.9–6.6)			
Procedure	6.5 (6.1–6.9)			
Control	6.7 (6.5–7.0)			
Pressure/tension (reversed)		0.96	0.58	0.47
Camera	3.0 (2.3–3.8)			
Procedure	3.4 (2.5–4.2)			
Control	3.0 (2.6–3.5)			

## Discussion

The results show that the technical aspects of camera navigation skills improve after simulation-based training, but we could not find a significant difference when examining transfer to the OR. This is in contrast to previous findings, which showed that simulation-based camera navigation skills transferred to the operating room, and this training was as effective as the traditional hands-on surgical training, while more time-efficient [11]. It can appear overwhelming for a surgical novice to assist during surgery for the first time, and the unfamiliar environment can assumedly compromise focus on the role as an assistant. Three participants experienced short episodes of lightheadedness, but all were able to complete the procedure.

A laparoscopic cholecystectomy is a commonly performed as an outpatient procedure and was chosen as the procedure to test camera navigation skills in the OR. However, the camera movement during a cholecystectomy is limited, and it is therefore less challenging to handle the scope compared with other procedures. A procedure such as a laparoscopic hernia repair requires more movement of the laparoscope, which could have provided a greater challenge and may be a better way to assess the camera navigator.

No significant difference was found between the camera group and the procedure group. Camera navigation was superior to no training in total time and total angular path length, while training on a surgical procedure was superior to no training only in total time. This suggests that training deliberately on camera navigation tasks is important when structuring a training program for camera navigation skills, and that basic skills such as instrument and scope handling should be mastered before continuing with more complicated tasks, such as procedures, especially because complications are more likely to occur due to poor camera navigation [4], and the total operative time increases when medical students are present during laparoscopic surgery [2]. These findings are confirmed by other studies done in a simulated setting on knot-tying skills, where both time and errors increased as the rotational effects of the camera increased [19–21].

Greater exposure to surgery as a medical student increases the likelihood of choosing a surgical career [22, 23], enables the practice of surgical skills, and therefore eases the transition from being an assistant to becoming the operating surgeon. Incorporation of motivational factors in medical education research could help understanding of the components of learning [12, 13]. Surprisingly, we found a significant difference on two dimensions (interest/enjoyment and perceived choice) on the IMI between the camera group and the control group. The findings indicate that the control group shows a higher level of interest/enjoyment and a higher level of perceived choice toward the task as an operating assistant, compared to the camera group. This can possibly be explained by the participants’ lack of expectations toward the task and their performance of the task in the control group. However, all three groups scored relatively high on this dimension, compared to a previous study [24]. The findings are surprising and oppose what was hypothesized. This trial is, to our knowledge, the first study to examine motivational factors in simulation-based laparoscopic training.

### Strengths and limitations of the trial

Due to time restriction, we used time-limited simulation training instead of proficiency-based and distributed training [25], which optimally should have been used. It is likely that the camera group managed to reach proficiency within the time frame, but less likely that the procedure group did, due to the more complicated nature of the procedural task. It is, however, difficult to compare interventions using different training content with a proficiency-based design.

The use of a previously validated assessment model [18] in the simulation test is a strength, as is the use of standardized pretest instructions by the same instructor before initiating the simulation test.

A strength of the transfer test is that the same two surgeons who either performed or supervised the procedure, also rated all participants. The assessment tool used was designed by an expert group but not previously validated, which is a limitation.

Even though we tried to stratify the randomization by sex, the distribution in the groups was not optimal. This could potentially influence the results, as men are more likely to perform better than women, especially during initial simulator training [26].

All but one fully complied with the trial; the one dropout resulted in not meeting the sample size in the camera group. The sample size was already small, but the dropout further increased the risk of Type II error.

With focus on efficiency and fewer working hours, the implementation of a training program in camera navigation and basic laparoscopic skills during medical school might be valuable, but further research into the relevance of simulation-based camera navigation training is necessary. It might be relevant to incorporate scenarios with suboptimal viewing conditions that could potentially prepare and/or help the surgeon to identify suboptimal viewing conditions, simultaneously helping the camera navigator understand the consequences of suboptimal viewing. Team training appears to be beneficial and has been shown to both shorten the learning curve and improve outcome. [27] During team training, the camera navigator and the surgeon simultaneously practice their skills, assumedly improving both technical and non-technical skills, the role of simulation-based camera navigation training in the context of team training would also be relevant to examine further.
